# A Variety of Nucleic Acid Species Are Sensed by cGAS, Implications for Its Diverse Functions

**DOI:** 10.3389/fimmu.2022.826880

**Published:** 2022-02-04

**Authors:** Dawei Wang, Heng Zhao, Yangkun Shen, Qi Chen

**Affiliations:** Fujian Key Laboratory of Innate Immune Biology, Biomedical Research Center of South China, Fujian Normal University, Fuzhou, China

**Keywords:** cGAS, STING, Interferon, Nucleic acid recognition, Disease treatment strategy

## Abstract

Cyclic GMP-AMP synthase (cGAS) recognizes double-stranded DNA (dsDNA) derived from invading pathogens and induces an interferon response *via* activation of the key downstream adaptor protein stimulator of interferon genes (STING). This is the most classic biological function of the cGAS-STING signaling pathway and is critical for preventing pathogenic microorganism invasion. In addition, cGAS can interact with various types of nucleic acids, including cDNA, DNA : RNA hybrids, and circular RNA, to contribute to a diverse set of biological functions. An increasing number of studies have revealed an important relationship between the cGAS-STING signaling pathway and autophagy, cellular senescence, antitumor immunity, inflammation, and autoimmune diseases. This review details the mechanism of action of cGAS as it interacts with different types of nucleic acids, its rich biological functions, and the potential for targeting this pathway to treat various diseases.

## Introduction

In the 1980s, researchers already understood the correlation between bacterial DNA and immune activation *in vitro* (1), i.e., they knew that exposing macrophages to bacterial DNA could stimulate the expression of interferon-alpha (IFN-α) and interferon-beta (IFN-β), and induce the activation of natural killer cells and the release of interferon-gamma (IFN-γ) (1, 2). Further studies showed that the immune activation induced by bacterial DNA is related to its large number of unmethylated CpG motifs, which are different from the DNA motifs in humans and mice (3, 4). When bacteria-derived DNA invades human cells, the host will recognize its pathogen-associated molecular patterns and trigger an immune response (3, 5, 6). Similarly, when a virus infects a host cell, the nucleic acid released into the cell triggers the intracellular immune response, causing antiviral immunity (7–9). Thus, identifying nucleic acids derived from pathogens is an important task for a host cell to mount an immune response and eliminate them.

Host cells have evolved different recognition patterns for different types of nucleic acids, *via* various pattern recognition receptors (PRRs). PRRs include mainly Toll-like receptors (TLR) (10), NOD-like receptors (NLRs) (11), C-type lectin receptors (CLRs) (12), RIG-I-like receptors (RLRs) (13), and DNA receptors/sensors (14). Among these, DAI (15), IFI16 (16, 17), DDX41 (18), MRE11 (19), LSm14A (20), DHX9 (21), hnRNPA2B1 (22), and cGAS (23–25) are considered DNA receptors/sensors.

cGAS is a cytoplasmic nucleic acid sensor with the widest recognition ability for double-stranded DNA (dsDNA); it binds dsDNA in a manner independent of sequence specificity (23). cGAS can also recognize other types of nucleic acids that trigger other important functions dependent on its subcellular location. The classic function of cGAS is to activate the downstream adaptor protein STING to induce IFN production and release. While the cGAS-STING signaling pathway plays a critical role in resisting pathogen invasion, excessive activation of this pathway can cause chronic inflammation, autoimmune disease, and cancer. Therefore, cGAS-STING signaling must be tightly regulated.

## cGAS-Mediated Signaling Pathways

**Figure 1:**
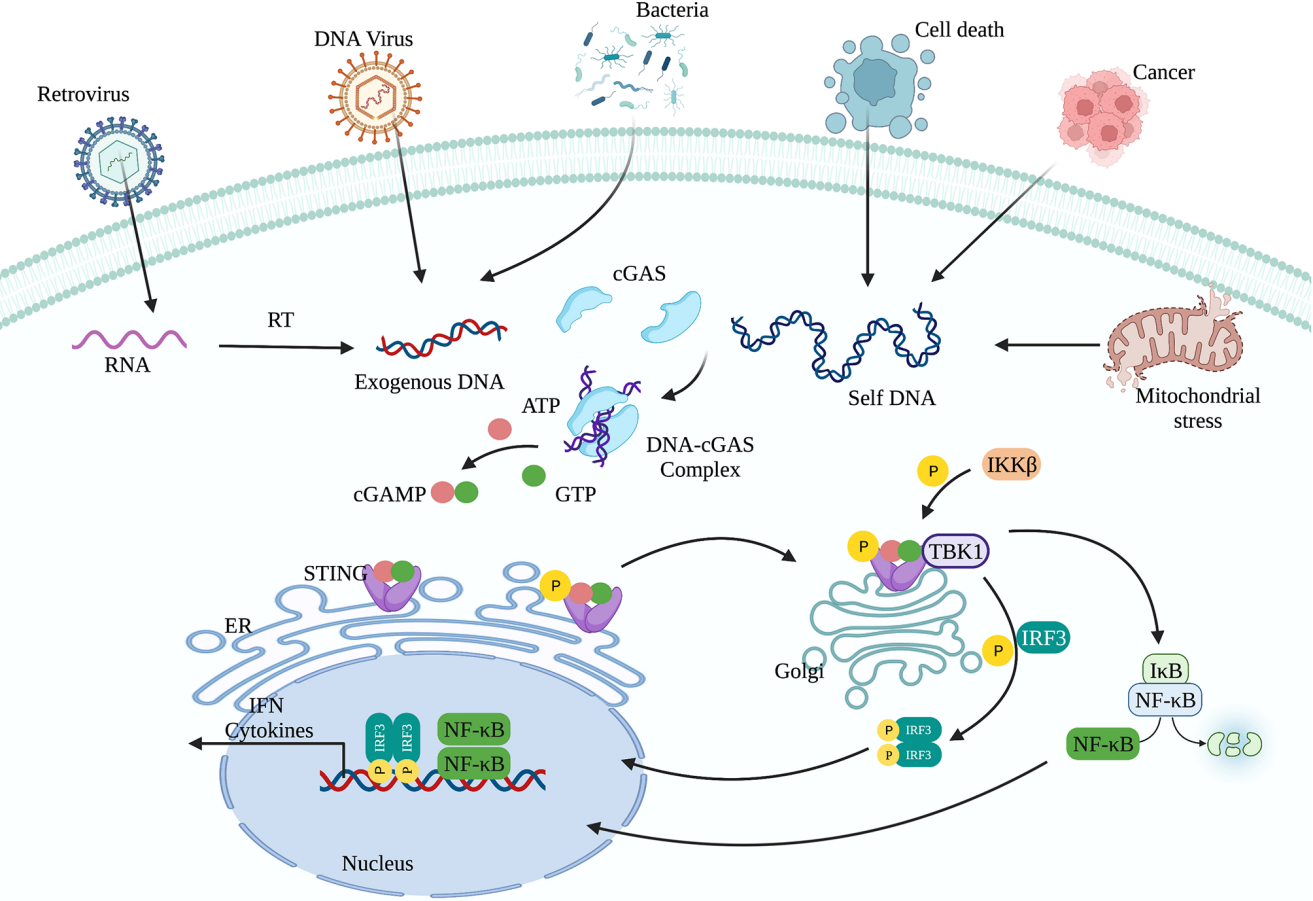
The cGAS-STING signaling pathway. cGAS can recognize exogenous DNA from pathogens or self-DNA (from the mitochondria and nucleus). After cGAS recognizes DNA, it synthesizes the second messenger 2’3’-cGAMP, which activates STING. The translocation of STING from the endoplasmic reticulum to the Golgi apparatus is a prerequisite for participating in the downstream signal transduction and regulating IFN-I transcription. Activated STING can recruit and activate IKK and TBK1. TBK1 phosphorylates IRF3. Subsequently, phosphorylated IRF3 forms a dimer and translocates to the nucleus, where it acts together with NF-κB to initiate the expression of IFN-I and other immune regulatory factors.

cGAS-STING signaling presents an evolutionarily highly conserved mechanism of immunity (26, 27). Upon recognition of cytoplasmic DNA, cGAS uses ATP and GTP as substrates to synthesize cyclin GMP-AMP (cGAMP), which act as a second messenger to activate STING (28, 29). Activation of STING is critical for initiating the downstream immune cascade (30). Translocation of STING from the endoplasmic reticulum (ER) to the Golgi apparatus is a prerequisite condition for downstream signal transduction and transcriptional regulation of type I interferons (IFN-I). STING activation requires the palmitoylation of Cys88 and Cys91, which takes place in the Golgi (31). Palmitoylation may also promote the oligomerization of STING and the activation of TANK-binding kinase 1 (TBK1). Activated STING can recruit and activate inhibitor of nuclear factor kappa-B kinase (IKK) and TBK1, but recruiting TBK1 is not sufficient to activate interferon regulatory factor 3 (IRF3) (32). Phosphorylation of STING at Ser366 by TBK1 allows it to interact with IRF3, and facilitate TBK1 phosphorylation of IRF3 (33). Phosphorylated IRF3 forms a dimer and transfers to the nucleus, where it acts together with nuclear factor-κB (NF-κB) to initiate the expression of IFN-I and other cytokines (**Figure 1**) (33, 34). After STING is activated, the “unfolded protein response (UPR) motif” at the C-terminus triggers the ER stress response and autophagy by activating the formation of the Unc-51-like autophagy activating kinase (ULK1) complex and the Beclin-1-class III phosphatidylinositol-3 kinase (PI3KC3) complex (35, 36). cGAS can also trigger autophagy through direct interaction with the Beclin-1-PI3KC3 complex (37). Autophagy mediated by the cGAS-STING pathway can spread to the whole cell, helping to remove pathogenic microorganisms (38) as well as excessive inflammatory factors and cytoplasmic DNA to prevent overactivation of the inflammatory response (39). Autophagy thus acts as negative feedback mechanism to limit continuous activation of the cGAS-STING signaling pathway.

### Structural Basis for cGAS Binding dsDNA

**Figure 2:**
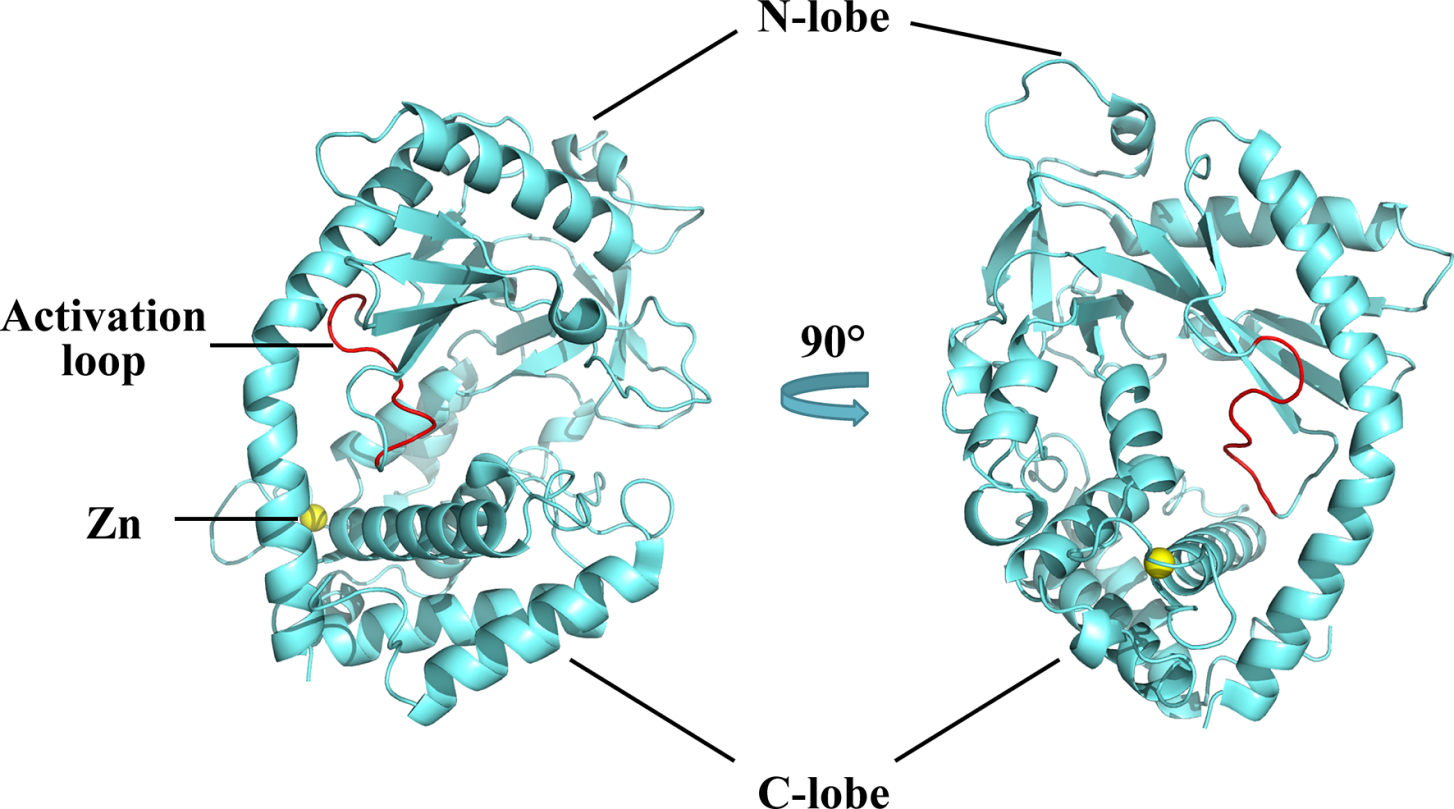
Structures of human cGAS in the apo state. Human cGAS consists of two lobes. The N-terminal lobe consists of two helices and a highly twisted beta-sheet, and the C-terminal lobe is a helix bundle, which contains a conserved zinc finger motif and a leucine residue. Between the two lobes is a large cleft, which is the catalytic site for substrate binding. In the apo state, cGAS maintains an autoinhibitory U-shaped conformation (Structure of human cGAS adopted from PDB:4KM5).

The structure of cGAS with or without bound dsDNA has been resolved in various species (40–44). cGAS is inactive and maintains a U-shaped conformation until DNA binding induces a conformational change that remodels the enzyme active site into activated state (42, 45). Human cGAS contains 522 amino acid residues and adopts the characteristic bi-lobal fold of the nucleotidyltransferase family (42). The N-terminal lobe is a non-structural, positively charged domain, which consists of two helices and a highly twisted beta-sheet; all catalytic residues are located on the central beta-sheet (46). The C-terminal lobe is a helix bundle that contains a conserved zinc finger motif and a leucine residue (41, 46). The zinc finger motif mediates DNA binding and cGAS dimerization (46), and the leucine residue acts as a conservative structural switch that strictly regulates cGAMP production in response to dsDNA binding (41). The cleft between the N- and C-terminal lobes constitutes the substrate binding site of the enzyme (**Figure 2**) (42). The C-terminal domain of cGAS is highly conserved whereas the amino acid sequence homology of the N-terminal domain is low, though the residues that play decisive functional roles are relatively conserved (47).

A positively charged patch, located in the groove on the backside of the substrate binding cleft, is the major DNA binding site in cGAS (44). The activation loop, containing residues 210 to 220, among which Gly212 and Ser213 are highly conserved, is located near the DNA binding surface of cGAS and undergoes a conformational change similar to a switch after DNA binding (42). Meanwhile, Asn210 is critical for the enzymatic activity of cGAS. Deamination of Asn210 affects cGAMP synthesis, but does not weaken the self-dimerization, dsDNA-binding, or nucleotide-binding activity of cGAS (48). Upon DNA binding, the DNA binding site clashes with the activation loop, causing the loop to move inward and expose the binding site to the donor substrate. In contrast, modeling of the cGAS structures bound to dsRNA shows that the activation loop inserts into the major groove of dsRNA and does not cause detectable conformational changes, which may explain why dsRNA is unable to activate cGAS (42). In addition to the conformational changes in the activation loop, DNA binding induces a reorganization of the two loops at the entrance of the catalytic site in the N-terminal lobe of cGAS (44). These conformational changes promote enzyme activation by configuring the active site for Mg^2+^ ion binding and enhancing accessibility of the active site to the substrate (46).

### cGAS-DNA Complex and Phase Separation

cGAS and DNA binding is primarily mediated by the interaction between positively charged residues on cGAS with the sugar-phosphate backbone of DNA, which explains the lack of sequence specificity of the interaction (46, 49). cGAS-DNA binding forms a droplet structure, resulting in phase separation, the degree of which can be enhanced by increasing the N-terminal binding valence (50). Through X-ray diffraction technology, it was found that long-stranded DNA can activate cGAS better than short-stranded DNA because the cGAS-DNA interaction forms a ladder-like complex; the longer the DNA chain, the more stable the structure of the complex, which implies a correlation between DNA length and binding efficiency (51). The formation of 2:2 cGAS-DNA complex is arranged into two DNA molecules in a roughly parallel manner, such that each complex has enhanced ability to bind subsequent cGAS dimers, thereby promoting a high degree of synergy in cGAS recruitment and activation (52). Once the cGAS-DNA complex is formed, a catalytic and accessible nucleotide-binding pocket is formed, the intermediate product pppGpA is synthesized, and subsequently cGAMP is generated (40).

### Cellular Localization of cGAS

cGAS was first identified as a cytoplasmic DNA sensor abundantly present in the cytoplasm of L929 and THP1 cells (23). The physical barrier between cGAS and self-DNA formed by the mitochondrial membrane and nuclear envelope is regarded as an important regulatory strategy that prevents self-DNA recognition and autoimmune activation. However, further research revealed additional subcellular localization of cGAS, including at the plasma membrane and in the nucleus (24, 25, 53–55). A recent study showed that cGAS is located in the plasma membrane through its N-terminal phosphoinositide binding domain, which selectively interacts with phosphatidylinositol 4,5-bisphosphate; cGAS lacking the N-terminal domain is mislocalized to the cytoplasmic and nuclear compartments (24). Membrane localization may help cGAS more rapidly detect viral DNA that enters cells through endocytosis, while also preventing cGAS from interacting with endogenous DNA (24). Nuclear localization of cGAS is found in epithelial cells, long-term hematopoietic stem cells (LT-HSC), and certain cancer cells (25, 54, 56, 57), and nuclear cGAS has additional functions (see below) (53, 55, 58, 59). Together, studies suggest that the cellular localization of cGAS varies greatly across cell types, which may be linked to specific functions of cGAS.

In addition, the cellular localization of cGAS appears to change during the cell cycle or under conditions of cellular stress. cGAS is mainly located in the cytoplasm during the interphase, and rapidly transfers to the chromosomes when the nuclear membrane disappears in metaphase (60). One study described that a gradual decrease in cGAS Y215 phosphorylation is accompanied by an increase in cGAS nuclear translocation in response to cell damage caused by DNA damaging agents (55). Another study found that in migrating mammalian cells, the nuclear membrane opens at a high frequency during interphase, which allows cytoplasmic cGAS translocation to chromatin (61).

## A Variety of Nucleic Acid Species Are Sensed by cGAS

### cGAS Recognizes Pathogen-Derived dsDNA

DNA immune recognition mediated by cGAS-STING signaling plays a vital role in preventing pathogenic microbial infection (62). cGAS recognizes pathogen-derived DNA to activate innate immunity and antiviral immune responses (63).

Herpes simplex virus 1 (HSV-1) is the first DNA virus shown to activate the cGAS-STING signaling pathway *in vitro* and *in vivo* (64, 65). *In vitro*, acetyltransferase KAT5 mediates cGAS acetylation upon HSV-1 infection, which enhances the affinity of cGAS binding to viral DNA and is thought to enhance antiviral immunity (66). *In vivo*, *cGas^-/-^* mice developed ataxia and paralysis and had a higher mortality rate upon HSV-1 infection; high titers of HSV-1 were also detected in the knockout mouse brain (67). This phenomenon has also been verified in *sting^-/-^* mice (68). In contrast, wildtype mice were less likely to develop symptoms or die after HSV-1 infection (67). In addition to HSV-1, Kaposi’s sarcoma-associated herpes virus (KSHV) can also activate the cGAS-STING signaling pathway (69). Compared with wildtype controls, cGAS or STING knockdown inhibited the expression of IFN-β in endothelial cells, and caused an increase in KSHV gene transcription and genomic copy number (69). DNA viruses such as human papillomavirus (70), cytomegalovirus (71), adenovirus (72), and vaccinia virus (73) have all been shown to activate cGAS and induce a host immune response to resist viral infection (74).

Many Gram-negative and positive bacteria can also activate the cGAS-STING signaling pathway. *Listeria monocytogenes* can replicate in the cytoplasm of human bone marrow cells, and its dsDNA is a major trigger of IFN-β expression dependent on IFI16, cGAS and STING (75). *Neisseria gonorrhoeae* induces the production of IFN through TLR4 and further enhances the IFN response by activating cGAS after it invades the cytoplasm of bone marrow-derived macrophages (76). In addition, the DNA derived from pathogenic microorganisms including *Mycobacteria*, *Legionella*, *Shigella*, *Francisella*, group B *streptococcus*, and *Chlamydia* can all be recognized by cGAS and activate STING to induce the body’s immune response to eliminate the invading pathogenic microorganisms (32).

Thus, recognizing pathogen-derived dsDNA by cGAS is a key event for the host to perceive pathogen invasion and induce a response. Although other PRRs can recognize pathogen-derived nucleic acids and activate an immune response, activation of the cGAS-STING signaling pathway plays an indispensable role in helping the host to resist pathogenic microorganism invasion.

### cGAS Recognizes Plasmid DNA

Plasmid transfection is used as a transient gene delivery system to express a foreign protein in the cell. Interestingly, a study found that after cells were transfected with foreign plasmids, the ability of host cells to prevent viral infections improved, suggesting that activation of cGAS-STING signaling may ready the cell for subsequently fighting viral infections (77).

In addition, our research group found that inhibition of cGAS by gene knockout or chemical inhibition can increase transgene expression at the transcriptional level, and that this increase is negatively correlated with IFN and interferon-stimulated gene (ISG) expression (78). Thus, targeting the cGAS-STING signaling pathway is likely an effective strategy for gene therapy and nucleic acid drug development.

### cGAS Recognizes Endogenous dsDNA

DNA is mainly stored in the nucleus; however, mitochondria, the organelles that supply energy to cells, also contain DNA molecules, namely mitochondrial DNA (mtDNA). Under normal circumstances, no (or little) free DNA is present in the cytoplasm and other organelles. Mitochondrial or nuclear damage caused by physical, chemical, and other factors can cause mtDNA or nuclear DNA to leak into the cytoplasm where it is recognized by cGAS, leading to the immune activation (59, 79, 80). Excessive activation of cGAS-STING signaling by endogenous dsDNA is related to the development of inflammatory and autoimmune diseases, including systemic lupus erythematosus (SLE), Aicardi-Goutières syndrome (AGS), and neurodegenerative diseases (81–83).

#### cGAS Recognizes dsDNA Derived From Mitochondria

Cells have many ways to maintain mitochondrial homeostasis. When mitochondria respond to stress, mtDNA is released into the cytoplasm through the Bax/Bak channel on the outer mitochondrial membrane. Subsequent activation of the mtDNA-cGAS-STING pathway induces the production of IFN-I (84).

**Figure 3:**
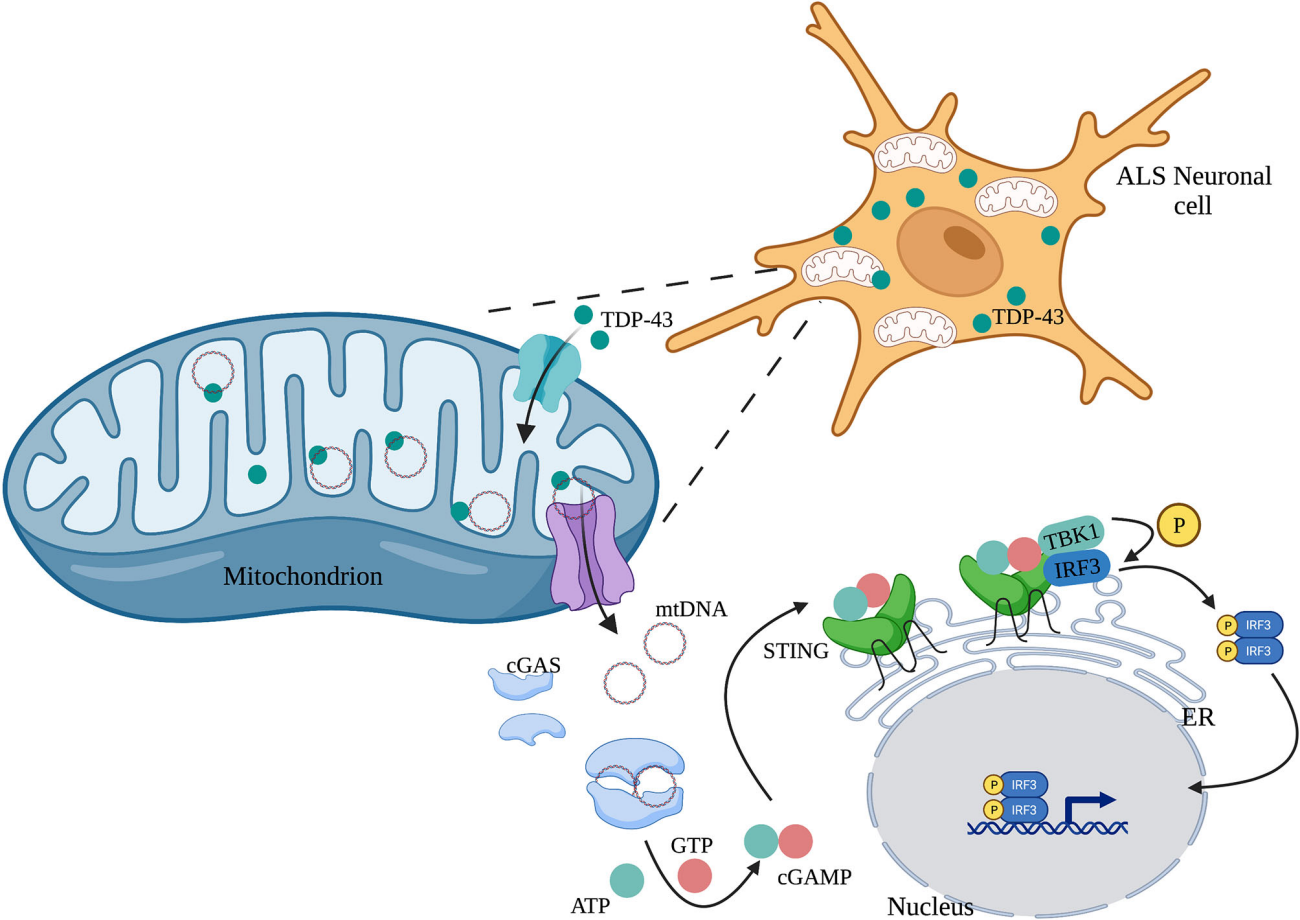
TDP-43 induces mtDNA leakage in ALS neuronal cells. A large amount of TDP-43 protein is present in the neuronal cells of patients with ALS, which causes mtDNA release into the cytoplasm through the mPTP pathway. mtDNA leakage in turn activates the cGAS-STING signaling pathway and induces the production of inflammatory factors.

Human mitochondrial transcription factor A (TFAM) is a type of mtDNA binding protein that controls mtDNA separation, abundance, and nucleoid structure (85). Genetic deletion of TFAM causes abnormal accumulation of mtDNA in the cytoplasm, leading to the activation of the cGAS-STING signaling pathway and production of IFN-I and ISGs (86). TDP-43, a nuclear DNA/RNA binding protein, is present in patients with Alzheimer’s disease (87) and amyotrophic lateral sclerosis (ALS) (88). The inflammatory response triggered by TDP-43 depends on the cGAS-STING signaling pathway. In ALS, after TDP-43 enters the mitochondria of neuronal cells, it triggers mtDNA release into the cytoplasm through the mitochondrial permeability transition pore (mPTP), leading to the release of inflammatory factors and IFN-I mediated by the cGAS-STING signaling pathway (**Figure 3**) (89). Mitochondrial DNA can also be released from dying cells, which then triggers release of pro-inflammatory factors and IFN-I that acts on hematopoietic stem cells and has a profound impact on cell function (90, 91).

A study conducted in a cohort of White adults found that plasma mtDNA levels gradually increased after the age of 50 years; levels of tumor necrosis factor-α (TNF-α) and interleukin-6 (IL-6) were positively associated with plasma mtDNA levels, suggesting a possible correlation between the level of blood mtDNA and age-associated mild chronic inflammation (92). Although the detailed mechanism is unclear, these pro-inflammatory and apoptotic factors may increase through the mtDNA-cGAS-STING pathway. In addition, the internalized bacterial endotoxin lipopolysaccharide activated Gasdermin D, which promotes the formation of mitochondrial pores and induces mtDNA release into the cytosol of endothelial cells (93). The released mtDNA was recognized by cGAS, leading to the synthesis of cGAMP, which suppressed endothelial cell proliferation by down-regulating the YAP1 signaling pathway (93). In the inflammatory lung injury model, cGAS deficiency can restore the regeneration capacity of endothelial cells, suggesting that targeting the Gasdermin D-activated cGAS-YAP signaling pathway may be a new strategy for restoring endothelial function after inflammatory injury (93). Furthermore, viral infection also induces mtDNA release, for example, cGAS senses the virus by detecting the release of mtDNA during dengue virus infection (94), and viroporin activity of influenza virus M2 is essential for mtDNA release into the cytosol in a MAVS-dependent manner (95). mtDNA stress promotes cGAS-dependent cytoplasmic mtDNA recognition, enhancing antiviral signaling and IFN-I responses during infection by activating STING-TBK1-IRF3 signaling (86).

Overactivation of the mtDNA-cGAS-STING axis is an important factor in inflammation caused by mtDNA leakage. Conversely, mtDNA released in response to stress in tumor cells induces autophagy-dependent ferroptosis through cGAS-STING signaling pathway activation (96). Therefore, regulating the activation of cGAS-STING in response to mtDNA leakage could be a disease treatment strategy.

#### cGAS Recognizes Nuclear-Derived dsDNA in the Cytoplasm

**Figure 4:**
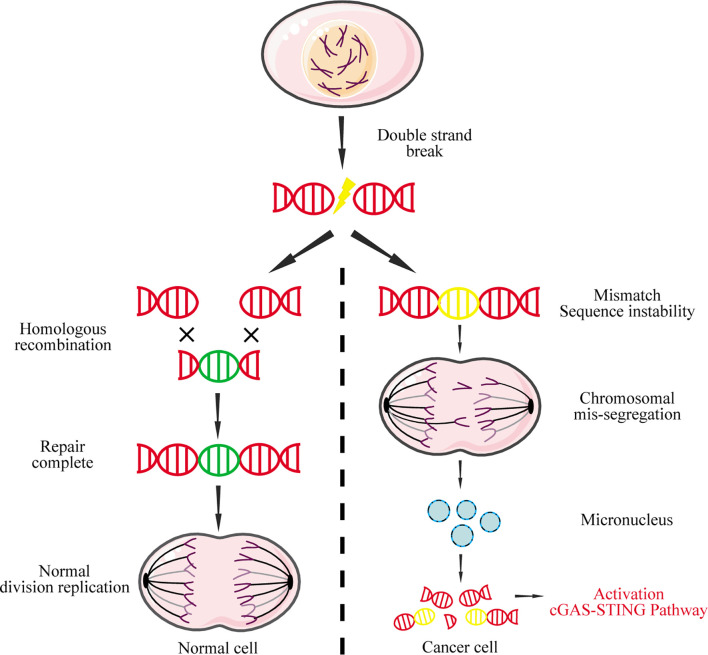
Micronucleus-derived DNA activates the cGAS-STING signaling pathway. DNA double-strand breaks caused by physical or chemical factors during mitosis can be repaired by homologous recombination in normal cells. However, due to the instability of the cancer genome, chromosomal missegregation is often caused during mitosis, resulting in the formation of micronuclei. The nuclear membrane of these micronuclei is fragile and easily ruptured, causing the DNA to leak into the cytoplasm, which in turn activates the cGAS-STING signaling pathway.

Nuclear DNA leakage, which forms micronuclei in the cytoplasm, is the main source for abnormal accumulation of cytoplasmic DNA (97). In normal cells, DNA double-strand breaks can be precisely repaired by homologous recombination to maintain genome stability and inhibit tumorigenesis (98). In contrast, widespread instability of the tumor cell genome could lead to chromosome pulverization that generates micronuclei during mitosis (99). The nuclear membrane of micronuclei is unstable and easy to rupture, causing micronucleus-derived DNA to activate cGAS and induce IFN-I (**Figure 4**) (100).

DNA damage and the expression of senescence-associated secretory phenotype (SASP) factors, including pro-inflammatory factors, are the key signs of cellular senescence (101, 102). Once cells enter the senescence process, nuclear membrane damage occurs, causing nuclear DNA leakage. The nuclear-derived DNA activates cGAS, which in turn increases SASP expression (101). Deletion of cGAS eliminates SASP gene expression and other cellular senescence markers, suggesting a key role of cGAS in regulating the effect of DNA damage, SASP expression, and cellular senescence (101).

#### cGAS Enters the Nucleus to Recognize Genomic DNA

cGAS has been reported to be abundantly present in the nucleus of certain cells, including HeLa and MEF cells (103), which raises two issues that need to be addressed. First, it is unclear whether there are functional differences between nuclear-localized cGAS and cytoplasmic cGAS. Second, if nuclear-localized cGAS can also recognize DNA, what mechanism is involved? Indeed, nuclear-localized cGAS displays non-canonical functions independent of STING. One study found that cGAS slows down the replication fork by interacting with replication fork proteins in a DNA binding-dependent manner (58). Another study revealed that nuclear cGAS recruits protein arginine methyltransferase 5 to the enhancer of antiviral genes and enhances antiviral gene transcription through histone modification, thereby inducing innate immune responses (53). In addition, during DNA damage, nuclear translocation of cGAS is induced in a manner that is dependent on importin-α (55). In the nucleus, cGAS is recruited to DNA double-strand breaks and interacts with PARP1 through poly (ADP-ribose), which hinders the formation of PARP1-Timeless complex and inhibits the homologous recombination of broken double strands to promote tumorigenesis (55). In addition to the above non-canonical functions, activation of the IFN pathway by cGAS is inhibited during mitosis. Recent research revealed a critical mechanism underlying cGAS inactivation in mitosis: nuclear cGAS is tethered tightly by a salt-resistant interaction, which maintains the quiescent state of cGAS and prevents autoreactivity (25). Barrier-to-autointegration factor 1 (BAF), a chromatin-binding protein, can also inhibit cGAS activity through competitive binding with dsDNA, thereby inhibiting the formation of cGAS-DNA complexes during mitosis (104). Another study found that nuclear cGAS binds to the negatively charged acidic plaques formed by histones H2A and H2B through its second DNA binding site, which blocks the binding of cGAS and dsDNA and maintains nuclear cGAS in an inactive conformation (103, 105). In addition, two studies revealed that during mitotic entry, the CDK1-cyclin B complex hyperphosphorylates human cGAS at S305 (or mouse cGAS at S291), which inhibits its ability to synthesize cGAMP. Upon mitotic exit, type 1 phosphatase dephosphorylates cGAS to restore its DNA sensing ability (60, 106).

In summary, cells utilize several ingenious molecular mechanisms to mitigate the potential immune activation caused by the recognition of nuclear DNA by cGAS, thereby ensuring cGAS perform its biological functions normally. When these regulatory mechanisms fail, cGAS misrecognition of nuclear DNA can lead to various cellular dysfunctional processes, including cellular senescence, inflammation, and tumorigenesis (17, 97, 107–109).

#### cGAS Associates With Telomeric DNA

Telomeres are protective structures at the end of chromosomes that gradually shorten with cell division (110). When telomeres are shortened to the limit, DNA damage signaling will be activated, triggering replicative senescence (111). A recent study showed that cGAS binds to telomeric/subtelomeric and recruits CDK1, which blocks the recruitment of RNF8 and avoids inappropriate DNA damage repair during mitosis (111). cGAS deficiency will cause chromosome end-to-end fusion between short telomeres to form dicentric chromosomes, hindering the initiation of cellular replicative senescence, resulting in genomic instability and increasing the probability of cell cancerization (111). In addition, telomere dysfunction leads to production of extrachromosomal DNA fragments that promote autophagy by activating the cGAS-STING signaling pathway (112).

Telomerase activity is significantly elevated in cancer cells. However, there is another mechanism to maintain extrachromosomal telomere repeats (ECTR) DNA sequence in cancer cells called alternative lengthening of telomeres (ALT) (113, 114). Studies have confirmed that induction of ECTRs in normal human fibroblasts activates the cGAS-STING signaling pathway, which in turn induces IFN-β production and leads to cell proliferation defect (114). Given that IFN-β has the function of activating immunity (115), *in vivo*, ECTR-induced IFN-β produced by ALT-induced cancer cells may exert anticancer functions. However, cGAS and STING expression are lost in most ALT cancer cell lines (114). Therefore, specific activation of the cGAS-STING signaling pathway in ALT-induced cancers may become a new therapeutic option.

### cGAS Recognizes cDNA

cGAS can also recognize cDNA (ssDNA) reverse-transcribed from HIV-1 virus, causing a cascade of immune responses and inducing IRF3 activation and IFN production; these effects are inhibited in the absence of or by knocking down cGAS-STING signaling (116, 117). A subsequent study found that ssDNA is a predominantly cytosolic DNA species in the early stage of HIV infection. Stem-loop structures in primary HIV-1 cDNA, similar to the Y-form structure, activate cGAS in a sequence-specific manner (118). These phenomena were also observed for other retroviruses, including HIV-2 (119), mouse leukemia virus, and simian immunodeficiency virus (116).

Hepatitis B virus (HBV) is an enveloped virus containing partially double-stranded DNA, belonging to the Hepadnaviridae family. The mechanism for induction of innate immunity in response to HBV has been controversial (120–122). However, a recent study showed that HBV RNA does not cause immune stimulation in immunologically active bone marrow cells, while naked HBV DNA can (123). It was shown that the relaxed circular DNA (rcDNA) produced during HBV replication can be recognized by cGAS, thereby inducing an immune response (123).

Long interspersed element-1 (LINE1) is a type of retrotransposon (124). In the human genome, the vast majority of LINE1 are silent, but their overactivation can cause a variety of age-related pathologies, such as neurodegenerative diseases and cancer (125). SIRT6 is an ADP-ribose transferase enzyme/deacetylase involved in the regulation of LINE1 (126, 127). In *sirt6^-/-^* mice, the activity of LINE1 and the levels of IFN-I were significantly increased along with many aging-related characteristics, including growth retardation and a significantly shortened lifespan (128). *In vivo*, the use of nucleoside reverse transcriptase inhibitors (NRTIs) to target LINE1 can significantly extend the lifespan of *sirt6^-/-^* mice; *in vitro*, inhibiting LINE1 with siRNA or NRTIs can eliminate IFN-I production. The number of detectable DNA damage markers in the cytoplasm is also significantly reduced (128). Interestingly, cGAS expression was also elevated in *sirt6^-/-^* MEF cells. Knockdown of cGAS in *sirt6^-/-^* MEF cells resulted in a decrease of IFN-I in the cytoplasm. Further evidence shows that cGAS can induce IFN-I by recognizing the cDNA transcribed from LINE1 (128).

### Potential Functional Relationships Between cGAS and RNA

#### Virus-Derived RNA

Current research shows that the expression of IFNs induced by cGAS is stimulated by dsDNA, not RNA binding. The induction of IFN-β by dsRNA analogs poly(I:C) and poly(dA:dT) depends on the classic RIG-I-like receptor, not cGAS (129). Sendai virus is a known RNA virus that activates the RIG-I pathway and induces IFN-β expression, which is not affected by cGAS or STING deletion (67). However, not all RNA viruses follow the classic receptor recognition pathway. Some RNA virus infections seem to be affected by cGAS. West Nile virus is a single-stranded RNA virus, but *cGas^-/-^* mice are significantly more susceptible to infection compared to wildtype controls (130). Chikungunya virus (CHIKV) is another positive-sense single-stranded RNA virus. A study reported that the CHIKV capsid protein could induce cGAS degradation, thereby inhibiting DNA-dependent IFN-β transcription, whereas the cGAS-STING signaling pathway restrained CHIKV replication in fibroblasts and immune cells (131). Therefore, cGAS deficiency may downregulate certain antiviral genes, making cells more susceptible to some RNA viruses.

Interestingly, cGAS can recognize DNA : RNA hybrids and efficiently synthesize cGAMP in THP1 cells, although the induced cGAMP is less than that induced by dsDNA (132). In one protein-nucleic acid interaction model, RNA : DNA hybrids could bind the cGAS cleft in the same way as dsDNA, regardless of the orientation of the RNA and DNA strands. The structural comparison of dsDNA and RNA : DNA hybrids shows that they have similar double-stranded helical conformations, and their small and large grooves have similar shapes (132). Therefore, the mechanism by which RNA : DNA hybrids activate cGAS may be similar to that used by dsDNA to activate cGAS. Though the cGAS-STING signaling pathway responds to some RNA virus infections, the detailed underlying mechanism needs further investigation.

#### Circular RNAs (circRNAs)

circRNAs are a widespread form of non-coding RNA in eukaryotes, with tissue-specific and cell-specific expression patterns, whose biogenesis is regulated by specific cis-acting elements and trans-acting factors (133).

Under homeostasis, most hematopoietic stem cells in the bone marrow are quiescent and maintain the potential for self-renewal and differentiation (134). Disrupting the balance between self-renewal and differentiation of hematopoietic stem cells can cause bone marrow failure or hematological malignancies (135). A recent study found a circRNA derived from the D430042O09Rik gene transcript in mice, cia-cGAS, regulates the long-term homeostasis of hematopoietic stem cells (57). cia-cGAS is highly expressed in the nucleus of LT-HSC. IFN-I expression is increased in the bone marrow of cia-cGAS deficient mice, which in turn causes hematopoietic stem cells to exit the G0 phase and enter the active phase until exhaustion (57). Under homeostasis, cia-cGAS binds to cGAS in the nucleus, inhibits its enzymatic activity, and protects LT-HSC in the dormant phase from cGAS-mediated “cell exhaustion”. Furthermore, the binding affinity of cia-cGAS to cGAS is stronger than self-DNA to cGAS, which inhibits the production of IFN-I mediated by cGAS in LT-HSC, thereby maintaining the steady-state of LT-HSC (57).

This study provided an interesting new avenue for exploring the correlation and interaction between cGAS and circRNA, suggesting a potential new function of cGAS that is distinct from its role as a DNA sensor.

## cGAS Signaling in Cellular Dysfunction

### cGAS Signaling and Cellular Senescence

Cellular senescence is a state of irreversible growth arrest caused by various factors including oxidative stress, oncogenic stress, and telomere shortening. Although the causes and phenotypes of cellular senescence are diverse, a persistent DNA damage response is considered to be an important feature of cellular senescence (136).

Multiple studies provide strong evidence that cGAS plays an important role in promoting cellular senescence (101, 137, 138). With successive passaging of primary MEFs, most of the cells eventually senesce, and only a small fraction overcome the growth crisis and become immortal. Indepth studies have found that cGAS deletion accelerates the spontaneous immortalization of MEF cells, because the absence of cGAS eliminates SASP induced by spontaneous immortalization or DNA damaging agents (101). In addition, cGAS is activated by cytosolic chromatin fragments in senescent cells, which triggers the production of SASP factors through STING, thereby promoting paracrine senescence (137). These studies provided new insight into the mechanism of cellular senescence by establishing the cGAS-STING signaling pathway as an intermediate bridge between senescence and the SASP.

### cGAS Signaling and Inflammation

The aberrant activation of the cGAS-STING signaling pathway has been implicated in a variety of inflammatory diseases.

Myocardial infarction (MI) involves a strong inflammatory response in related tissues. One study found that cGAS activation by self-DNA from apoptotic cells is the main cause of MI-related IFN-I production (139). In this model, ischemic myocardial injury causes cardiomyocyte damage and nucleic acid release, which activates the cGAS-STING axis. Compared to wildtype littermates, the survival rate of cGAS-deficient mice after MI is significantly higher, with the mice also showing reduced pathological remodeling including ventricular rupture, enhanced angiogenesis, and maintenance of myocardial contractile function (140).

Aicardi-Goutières syndrome (AGS) is a rare genetic disease characterized by systemic inflammation that most commonly affects the brain and skin. Patients with this disease often develop severe physical and mental disorders, chronic aseptic lymphocytosis, and elevated IFN-I levels (82). Studies have confirmed that the loss-of-function mutation of trex1 exonuclease is related to the development of AGS (141, 142). Similar to human AGS patients, trex1-deficient mice develop autoimmune disorders and fatal inflammatory phenotypes associated with high expression of ISGs (143, 144), which can be rescued by cGAS gene knockout (145). Elevated cGAMP can be detected in tissues from *trex1^-/-^* mice, demonstrating that cGAS is activated in these mice (142).

Systemic lupus erythematosus (SLE) is a serious chronic inflammatory disease that can affect most of the body’s tissues and organs, including the skin, joints, kidneys, blood cells, and nervous system. Although the phenotype and course of SLE vary greatly, the disease is associated with a systemic increase of IFN-I and a defect in apoptotic cell clearance (81). A recent study showed that the expression of cGAS in peripheral blood mononuclear cells of patients with SLE was significantly higher than that of the control group; the higher the cGAMP level, the higher the disease activity in patients with SLE (146). Loss of trex1 can lead to accumulation of cytoplasmic DNA and the development of autoimmune diseases, including AGS and SLE. Our research group constructed *trex1^D18N/D18N^* mice, which show similar disease phenotypes as in patients with AGS and SLE. In these mice, we verified that cGAS deletion reduces multiple organ inflammation (147). Together, ours and others’ studies indicate that cGAS is a key mediator of autoimmune diseases related to trex1 dysfunction.

In conclusion, several studies strongly support a central role of the cGAS-cGAMP-STING pathway in the pathogenesis of various IFN-I-mediated inflammatory diseases. Therefore, the development of drugs that target this pathway may provide new hope for the treatment of such inflammatory diseases.

### cGAS Signaling and Tumorigenesis

The link between DNA damage and cancer has long been established. While this means that cells with DNA damage will be recognized and eliminated by immune cells, genomic instability itself is an important driver of cancer. Given the importance of cGAS in the DNA recognition pathway, it plays a crucial role in both aspects of cancer. Although the activation of the cGAS-STING signaling pathway has been tested as a potential cancer immunotherapy (see *Therapeutic Strategies in Tumor Immunotherapy*), the potential negative tumorigenic effects of overactivation of the cGAS signaling cannot be ignored.

7,12-dimethylbenz[a]anthracene (DMBA) is a known carcinogen. It activates the cGAS-STING signaling pathway by inducing DNA breaks and promotes skin carcinogenesis in mice. Interestingly, unlike other cancer models, DMBA-treated *sting^-/-^* mice were found to be more resistant to DMBA-induced skin cancer growth (148). Brain metastatic cells contain cytoplasmic dsDNA, which activate cGAS and produce large amounts of cGAMP. The connexin 43-based functional gap junctions between cancer cells and astrocytes allow the transfer of cGAMP to astrocytes, where it activates TBK1 and IRF3 and induces the production of IFN-α and TNF-α. These cytokines activate STAT1 and NF-κB signaling pathways in brain metastatic cells to support the growth and survival of cancer cells under the pressure of chemotherapy (149). Furthermore, the DNA damage caused by etoposide, camptothecin, and H_2_O_2_ treatment can induce nuclear translocation of cGAS. The nuclear cGAS significantly suppresses the repair of DNA damage mediated by homologous recombination, and induces transformation of the damaged cells leading to tumorigenesis (55).

## Therapeutic Strategies Targeting the cGAS Pathway

### Therapeutic Strategies in Inflammatory Diseases

Given that the cGAS pathway is involved in a variety of inflammatory diseases, inhibitors or antagonists targeting the cGAS-STING signaling pathway are being considered as potential therapeutics. At present, a variety of effective inhibitors/antagonists of cGAS have been developed. 2-amino pyridine ring (G150) blocks the binding of dsDNA and cGAS by occupying the ATP and GTP binding active sites on cGAS (150). Suramin replaces the DNA bound to cGAS to block the downstream immune response. *In vitro*, adding suramin to THP1 cells can effectively reduce the expression levels of IFN-β mRNA and protein (151). RU.521 selectively binds to cGAS, thereby inhibiting cGAMP induced by dsDNA and reducing expression of IFN in a dose-dependent manner, without affecting other inflammatory pathways independent of the cGAS pathway (152). Aspirin is a common non-steroidal anti-inflammatory drug. It can acetylate cyclooxygenase, including cGAS, thereby inactivating cGAS. One study confirmed that aspirin can effectively inhibit autoimmunity induced by self-DNA in the cells of patients with AGS and AGS mouse models (153).

### Therapeutic Strategies in Tumor Immunotherapy

Radiotherapy is a conventional cancer treatment method; damage of cancer cells triggers the release of pro-inflammatory factors and increases the infiltration of immune cells in the tumor (154). Studies have found that radiotherapy induces IFN-I in tumors and IFN-I receptors on immune cells (especially CD8^+^ T cells), which are critical to its therapeutic effectiveness (155, 156). Meanwhile, cGAS deficiency in dendritic cells (DC) is sufficient to eliminate antitumor immunity *in vitro* (157). Subsequent studies have shown that the cGAS-STING signaling pathway is an important contributor to antitumor immunity after radiotherapy by detecting DNA damage in tumor cells and promoting the recognition of tumor-derived DNA in immune cells (158, 159). Additional studies have shown that cGAS is essential for the antitumor effect of immune checkpoint blockade in mice (160). Antibodies against the immune checkpoint inhibitor PD1/PD-L1 can effectively slow the growth rate of mouse B16 melanoma (161). Intramuscular injection of cGAMP also inhibits the growth of melanoma and prolongs the survival of tumor-bearing mice, as cGAMP activates DC and enhances the cross-presentation of tumor-associated antigens to CD8 T cells. The combination of the PD-L1 antibodies and cGAMP has a synergistic effect beyond each treatment alone (160). These studies show that the activation of the cGAS pathway is important in anti-tumor immunotherapy; however, given that the cGAS pathway also has a tumor-promoting effect, unchecked activation of the cGAS signaling pathway in tumor cells is not a therapeutic option.

## Conclusions and Future Directions

**Figure 5:**
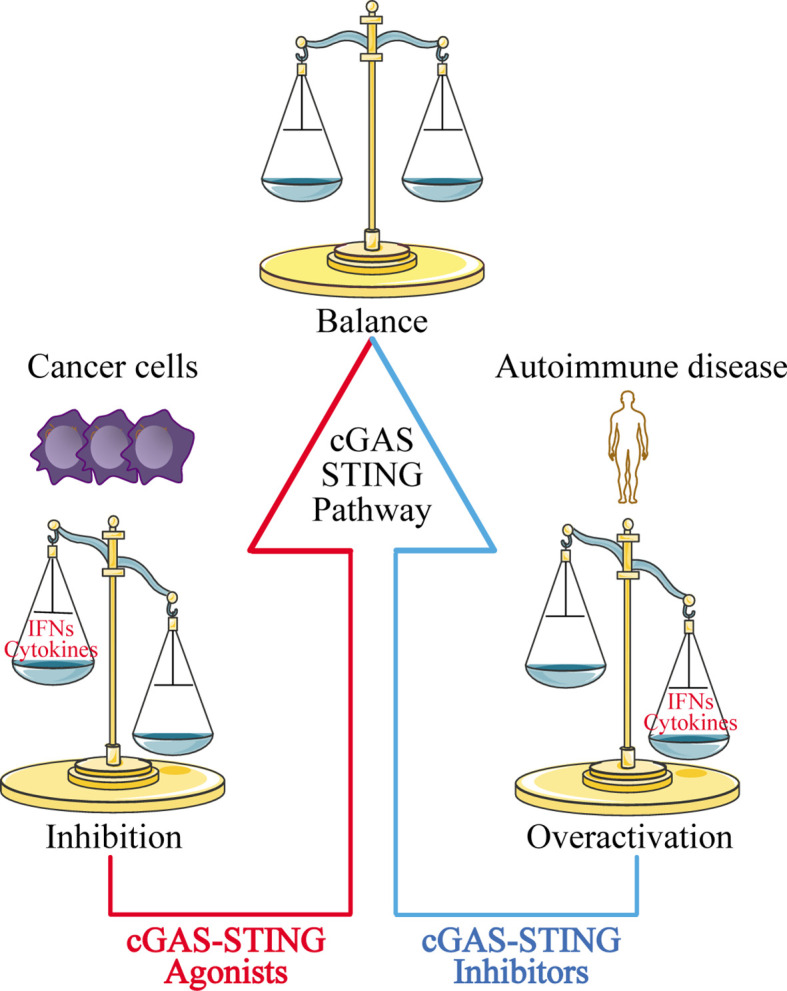
Disease treatment strategies targeting the cGAS-STING signaling pathway. For cancer cells with low expression of cGAS-STING, using cGAS-STING agonists to activate the pathway is one approach to restoring immune surveillance and enhancing immune cell infiltration into tumors. For autoimmune diseases and inflammatory diseases caused by overactivation of the cGAS-STING signaling pathway, blocking the pathway with cGAS-STING inhibitors may provide an effective therapeutic strategy.

The discovery of the cGAS-STING signaling pathway provides a comprehensive functional network for activation of the dsDNA-dependent innate immune response, and plays a particularly important role in activating the immune response against DNA viruses. The cGAS-STING signaling pathway also modulates cell transfection and gene delivery, and may be harnessed to enhance the development of new antiviral therapies and nucleic acid vaccines (68, 162). However, the recognition of self-DNA by cGAS can cause various diseases, including inflammatory and autoimmune diseases, largely due to the overexpression of IFN. Therefore, inhibitors targeting cGAS may be a promising treatment approach for such diseases. Given that STING is the most important adaptor protein mediating cGAS-mediated IFN expression, inhibitors targeting STING may also have therapeutic applications (**Figure 5**).

At the same time, researchers have also found that the cGAS-STING signaling pathway is inhibited in various cancer cells ranging from melanoma (163) to ovarian cancer (164), and colorectal carcinoma (165). The mechanisms underlying this phenomenon have yet to be uncovered. Our group recently used zebularine (a demethylating agent) to activate the cGAS-STING signaling pathway in tumor cells (166). In our mouse tumor models, zebularine enhanced STING expression by reducing DNA methylation on the STING gene promoter. Administration of zebularine alone reduced tumor burden and extended mouse survival; its combination with cGAMP or immune checkpoint inhibitors had a synergistic anti-tumor effect (166). Thus, activating the cGAS-STING signaling pathway in tumor cells can significantly enhance tumor immunotherapy effects (**Figure 5**).

In addition, the ability of cGAS to sense cDNA, DNA : RNA hybrids, and circRNA, as well as the functional differences related to its subcellular localization, indicates that cGAS has multifaceted biological functions. Although no research has shown whether dsDNA, cDNA, DNA : RNA hybrids, and circRNA have similar characteristics, it is conceivable that the nucleic acid-sensing ability of cGAS may depend on the modulation of its structural flexibility and the interaction between cGAS and the elaborate structures of these different nucleic acid species.

In summary, research on cGAS has expanded our understanding of its roles, beyond a traditional cytoplasmic nucleic acid sensor, and has clarified the mechanisms that cGAS uses to recognize different types of nucleic acids. These studies have shed light on the relationship between cGAS and antiviral immunity, tumor immunity, inflammatory response, and autoimmune diseases. The cGAS-STING signaling pathway may be a promising drug target for inflammatory and autoimmune diseases or inform the design of effective nucleic acid drugs to treat various diseases.

## Author Contributions

DW and YS conceived the manuscript. DW and HZ drafted the manuscript. QC revised the manuscript. All authors contributed to manuscript revision, read, and approved the submitted version.

## Funding

This work was supported by the National Natural Science Foundation of China (Grant No. 81770222).

## Conflict of Interest

The authors declare that the research was conducted in the absence of any commercial or financial relationships that could be construed as a potential conflict of interest.

## Publisher’s Note

All claims expressed in this article are solely those of the authors and do not necessarily represent those of their affiliated organizations, or those of the publisher, the editors and the reviewers. Any product that may be evaluated in this article, or claim that may be made by its manufacturer, is not guaranteed or endorsed by the publisher.
